# 3D Printed Personalized Nerve Guide Conduits for Precision Repair of Peripheral Nerve Defects

**DOI:** 10.1002/advs.202103875

**Published:** 2022-02-18

**Authors:** Kai Liu, Lesan Yan, Ruotao Li, Zhiming Song, Jianxun Ding, Bin Liu, Xuesi Chen

**Affiliations:** ^1^ Department of Hand and Foot Surgery The First Hospital of Jilin University 1 Xinmin Street Changchun 130061 P. R. China; ^2^ Key Laboratory of Polymer Ecomaterials Changchun Institute of Applied Chemistry Chinese Academy of Sciences 5625 Renmin Street Changchun 130022 P. R. China; ^3^ Biomedical Materials and Engineering Research Center of Hubei Province State Key Laboratory of Advanced Technology for Materials Synthesis and Processing Wuhan University of Technology 122 Luoshi Road Wuhan 430070 P. R. China; ^4^ Department of Sports Medicine The First Hospital of Jilin University 1 Xinmin Street Changchun 130061 P. R. China; ^5^ State Key Laboratory of Molecular Engineering of Polymers Fudan University 220 Handan Road Shanghai 200433 P. R. China

**Keywords:** peripheral nerve repair, personalized nerve guide conduit, precision tissue regeneration, three‐dimensional printing, tissue engineering

## Abstract

The treatment of peripheral nerve defects has always been one of the most challenging clinical practices in neurosurgery. Currently, nerve autograft is the preferred treatment modality for peripheral nerve defects, while the therapy is constantly plagued by the limited donor, loss of donor function, formation of neuroma, nerve distortion or dislocation, and nerve diameter mismatch. To address these clinical issues, the emerged nerve guide conduits (NGCs) are expected to offer effective platforms to repair peripheral nerve defects, especially those with large or complex topological structures. Up to now, numerous technologies are developed for preparing diverse NGCs, such as solvent casting, gas foaming, phase separation, freeze‐drying, melt molding, electrospinning, and three‐dimensional (3D) printing. 3D printing shows great potential and advantages because it can quickly and accurately manufacture the required NGCs from various natural and synthetic materials. This review introduces the application of personalized 3D printed NGCs for the precision repair of peripheral nerve defects and predicts their future directions.

## Graphical abstract

The personalized nerve guide conduits are quickly and accurately manufactured from various natural and synthetic materials by a three‐dimensional printing technique, which meets personalized imaging data to repair nerve defects of complex anatomical structures with the assistance of different growth factors and cells.

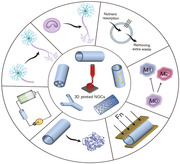

## 1 Introduction

Peripheral nerve injury (PNI) is a common disease that always results in loss of sensory and motor function.^[^ ^1^ ^]^ PNI is often caused by violent behaviors, motor vehicle accidents, and iatrogenic injuries.^[^ ^2^ ^]^ Generally, peripheral nerves have a solid ability to regenerate after injury.^[^ ^3^ ^]^ In the peripheral nervous system, axonal sprouting is initiated after the removal of myelin and axonal debris by Schwann cells (SCs) and macrophages.^[^ ^4^ ^]^ The regenerated axon reaches the distal end of nerve defect and recombines with the distal target.^[^ ^4^ ^]^ Surgery is the most common treatment strategy for PNI,^[^ ^5^ ^]^ which mainly includes neurorrhaphy, nerve grafting (autografts and allografts), and tissue engineering techniques. For nerve transections and short nerve defects (<1 cm),^[^ ^5^ ^]^ neurorrhaphy is a typical treatment method.^[^ ^6^ ^]^ Nerve autografting is currently the standard procedure for repairing nerve defects, which exceed 3 cm.^[^ ^6^, ^7^ ^]^ However, nerve autografting has many inevitable shortcomings, such as the limited donor, loss of donor function, formation of neuroma, nerve distortion or dislocation, and nerve diameter mismatch.^[^ ^8^ ^]^ In addition, decellularized allografts require the long‐term application of immunosuppressants, which may increase the risk of infection.^[^ ^9^ ^]^

**Scheme 1:**
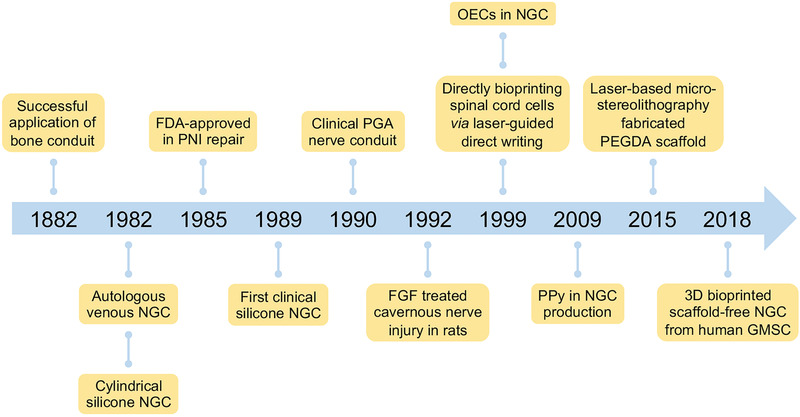
Historical timeline of momentous events in development of NGCs.

**Scheme 2:**
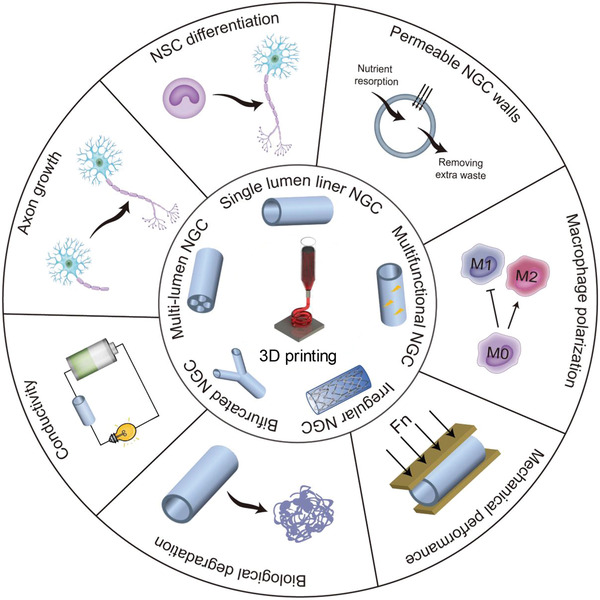
3D printed NGCs for nerve regeneration. Various types of NGCs with different topological structures and physical properties are manufactured by 3D printing for effective repair of nerve defects with complex anatomical structures. Reproduced with permission.^[^ ^31^ ^]^ Copyright 2019, Wiley‐VCH.

As a result, the limited functional recovery after treatments with autografts and allografts has promoted researchers to develop the nerve guide conduit (NGC) as an alternative.^[^ ^9^, ^10^ ^]^ The historical timeline of momentous events in the development of NGCs is shown in **Scheme** **1**.^[^ ^11^ ^]^ The design criteria for NGCs mainly include the following issues: 1) the suitable mechanical property; 2) the permeable conduit wall; 3) the low immunogenicity; 4) the adequate biodegradability (**Scheme** **2**).^[^ ^12^ ^]^ NGCs also need biomimetic conductive flexible integrated structures.^[^ ^6^ ^]^ Furthermore, NGCs can also enhance the therapeutic effects of peripheral nerve defects by combining other methods.^[^ ^13^ ^]^ Therefore, NCG has attracted increasing attention in recent years.

The traditional techniques used to fabricate NGCs mainly include solvent casting,^[^ ^14^ ^]^ phase separation,^[^ ^15^ ^]^ gas foaming,^[^ ^16^ ^]^ freeze‐drying,^[^ ^17^ ^]^ and electrospinning.^[^ ^18^ ^]^ However, these techniques have inherent limitations, as summarized in **Table** **1**.^[^ ^19^ ^]^ In contrast, the emerged three‐dimensional (3D) printing has revolutionized NGC manufacturing in recent years.^[^ ^20^ ^]^ 3D printing can effectively overcome the limitations of traditional techniques to produce NGCs with surface permeability while incorporating the needed cells and factors.^[^ ^19^ ^]^ 3D printed NGCs can simulate the structure and function of peripheral nerves with various biomaterials, biomolecules, and cells. In addition, 3D printing can also create personalized NGCs for patients through bioimaging and biomanufacturing technologies.^[^ ^21^ ^]^ In the following sections, we will systematically summarize the principles, material matrices, design features, performances, and advantages and disadvantages of different types of 3D printed NGCs for peripheral nerve regeneration (PNR). Finally, we will discuss the possible future research directions.

**Table 1:** Limitations of various NGC fabricating techniques

Fabricating technique	Limitation	Reference
Electrospinning	Poor repeatability and customizability; random and highly disordered fiber production	^[^ ^18^ ^]^
Freeze drying	Irregularly shaped pore	^[^ ^17^ ^]^
Gas foaming	Non‐porous external surface; poor interconnectivity	^[^ ^16^ ^]^
Phase separation	Material is limited to specific polymer	^[^ ^15^ ^]^
Solvent casting	Highly toxic solvent; low porosity (<50%); irregularly shaped pore	^[^ ^14^ ^]^
Common limitations	Inability to control porosity, pore size, and interconnectivity of NGC; poor repeatability; no determination of multilayer structure	^[^ ^19^ ^]^

Abbreviations: NGC, nerve guide conduit.

## 2 3D Printed Nerve Guide Conduits with Personalized Architectures

3D printing refers to accurately stacking printing materials layer by layer under the control of a computer to produce the structure of arbitrary shapes quickly.^[^ ^22^ ^]^ It is widely used in different fields, such as scientific research, aerospace, automobile manufacturing, healthcare, fashion, construction, and food industry.^[^ ^23^ ^]^ Although it is still in its infancy, it has shown great potential in promoting PNR.^[^ ^24^ ^]^ The selection of biomaterials for 3D printing can be summarized with the following primary considerations:^[^ ^25^ ^]^ 1) specific printability requirements; 2) biocompatibility and bioactivity;^[^ ^26^ ^]^ 3) appropriate physicochemical properties, mechanical strength, structural stability, and degradation kinetics;^[^ ^27^ ^]^ 4) affordability and regulation permission. The materials that have been used for PNR are summarized in **Table** **2**.^[^ ^27^, ^28^ ^]^

**Table 2:** Materials for making 3D printed NGCs

Type	Subtype	Material	Pros	Cons	Reference
Natural materials	_	Chitosan, collagen, gelatin, HA, silk fibroin	Biocompatible; combinable with synthetic materials; cross‐linkable; ease of degradability by natural enzymes; low immunogenicity	Low mechanical strength; rapid degradation in vivo	^[^ ^24^ ^]^
Synthetic materials	Conductive	CNT, graphene, PANI, PPy	Electrical stimulation of regenerating neurons; low immunogenicity; strong mechanical properties	Low cell adhesion; slow biodegradability	^[^ ^20^ ^]^
	Degradable	PCL, PGA, PGC, PHB, PLA, PLC, PLGA, PU	Low immunogenicity; tunable degradation rates, mechanical and swelling properties	The degradation of PLA/PLGA results in lactic acid by‐products, which may be neurotoxic at high concentrations	^[^ ^24^, ^29^ ^]^
	Non‐degradable	PE, PHEMA‐MMA, PPy, PVA, silicone	High mechanical strength; low immunogenicity	Chronic inflammatory response; increased swelling; sometimes reoperation	^[^ ^24^, ^32^ ^]^
	Hybrid	Chitosan, collagen, PCL, PGA, PHBV‐PLGA, PU, PVA	Exhibit favorable traits of polymers; generation of multi‐phase material matrix composites; optimized for bioprinting processability	Homogeneous inks cannot meet the demand for differences in material viscosities; multiple printheads may be required	^[^ ^37^ ^]^

Abbreviations: 3D, three‐dimensional; CNT, carbon nanotube; HA, hyaluronic acida; NGC, nerve guide conduit; PANI, polyaniline; PCL, poly(ε‐caprolactone); PE, polyethylene; PGA, poly(glycolic acid); PGC, poly(glycolide‐*co*‐ε‐caprolactone); PHB, poly(3‐hydroxybutyrate); PHBV, poly (3‐hydroxybutyrate‐*co*‐3‐hydroxyvalerate); PHEMA‐MMA, poly(2‐hydroxyethyl methacrylate‐*co*‐methyl methacrylate); PLA, poly(lactic acid); PLC, poly(L‐lactide‐*co*‐ε‐caprolactone); PLGA, poly(lactic‐*co*‐glycolic acid); PPy, polypyrrole; PU, polyurethane; PVA, poly(vinyl alcohol).

The bioink plays a significant role in the development of 3D bioprinting. In recent years, the development of bioink has made tremendous progress.^[^ ^24^, ^29^ ^]^ Nevertheless, most of the current bioinks are prepared by simply mixing hydrogel materials with cells and factors.^[^ ^30^ ^]^ In contrast to traditional methods, the 3D bioprinting integrates living cells into bioinks and prints them as cell‐loaded NGCs.^[^ ^13^ ^]^ The regenerative potential can be further enhanced by incorporating neurotrophic factors (NTFs) and drugs in combination with stem cells into the NGCs.^[^ ^12^ ^]^

**Figure 1:**
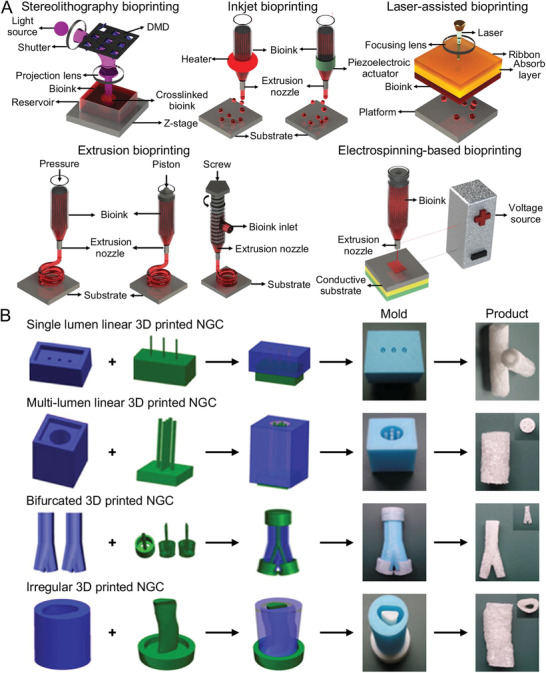
Strategies and products of 3D bioprinting. A) Schematic diagrams of different strategies currently used in 3D bioprinting. B) Indirect 3D Printing of NGCs: Single lumen linear NGA, multi‐lumen linear NGC, bifurcated NGC, irregular NGC. A) Reproduced with permission.^[^ ^31^ ^]^ Copyright 2019, Wiley‐VCH. B) Reproduced with permission.^[^ ^12^ ^]^ Copyright 2018, Elsevier Ltd.

In the past decades, various 3D bioprinting strategies brought new ideas to design medical implants (**Figure** **1**A).^[^ ^31^ ^]^ Each approach has its advantages and shortcomings (**Table** **3**). Each technique can be used to build 3D products, but each handles materials differently in the printing process (Figure 1B).^[^ ^12^ ^]^ Nevertheless, 3D bioprinting technology has demonstrated potential for preparing various types of NGCs.^[^ ^24^, ^32^ ^]^ It can also build individualized structures and even bind to different cells and nerve growth factors (GFs) in a single process to promote nerve growth in the right direction.^[^ ^27a^ ^]^ Regarding the design of 3D printers and classification of conventional 3D printing or bioprinting technologies, interested readers can refer to reviews by Cui,^[^ ^13^ ^]^ Dixon,^[^ ^12^ ^]^ Heinrich,^[^ ^31^ ^]^ Hutmacher,^[^ ^33^ ^]^ Joung,^[^ ^3^ ^]^ Maiti,^[^ ^22d^ ^]^ Sears,^[^ ^34^ ^]^ and Sachlos et al.^[^ ^35^ ^]^ In the following sections, we will introduce the characteristics of different types of NGCs and summarize them in **Table** **4**.

**Table 3:** Advantages and limitations of different 3D bioprinting technologies

Technology	Advantage	Limitation
Electrospinning‐based bioprinting	High resolution; optimal for fabrication of irregular NGCs	Complex operating systems and experimental procedures; high costs
Extrusion‐based bioprinting	A variety of materials are applicative; high viscosity and high cell density of bioinks; low cost and comparatively simple printed process	Anisotropic products; low printing speed and low precision; product resolution and cell viability depend on device
Inkjet bioprinting	High resolution, high accuracy, and high cell viability; mixed printing of multiple materials and colors; no waste of model materials and low cost; simple printing method	Limited to cell density and viscosity of bioinks; post‐processing can sometimes destroy some details; waste of supporting materials
Laser‐assisted bioprinting	High cell viability; high product resolution; suitable for all viscosities of bioinks	Bioink cell density limited; complex operating systems; high costs
Stereolithography	High resolution and high cell viability; simultaneous cross‐linking of entire layer and high production speed; wide range of applicable bioinks	Bioink cell density limited; complex operating systems; photoinitiator needs to be added; require transparent and photosensitive bioinks

Abbreviations: 3D, three‐dimensional; NGC, nerve guide conduit.

**Table 4:** 3D printed NGCs for never regeneration

Type	Fabrication method	Biomaterials and cell	In vitro/in vivo	Study period	Study result	Reference
Single lumen linear NGCs	3D printed molds	Gelatin cryogel, NIH‐3T3 cells, small molecule substance	Transected sciatic nerve in rat	3 months	NGCs significantly benefitted the recovery of transected peripheral nerve	^[^ ^51^ ^]^
	3D printed molds	EHS, GelMA	10 ‐mm sciatic nerve gap in rat	4 months	NGCs could promote the repair of peripheral nerve	^[^ ^44^ ^]^
	Layer‐by‐layer depositing, direct soaking	dECM, PCL, PDA	Cell behaviors and neuronal differentiation were assessed in vitro	−	NGCs could promote regeneration of nerve	^[^ ^58^ ^]^
	Assembling spheroids for constructing NGCs	NHDF	10 ‐mm nerve gap in rat	8 weeks	NGCs could enhance peripheral nerve regeneration	^[^ ^84^ ^]^
	Assembling spheroids for constructing NGCs	Canine dermal fibroblasts, silicon tube	5 ‐mm ulnar nerve gap in dog	10 weeks	NGCs were effective for nerve regeneration	^[^ ^50^ ^]^
	Stereolithography and coaxial electrospraying techniques	PC‐12 neural cells, PEG, PEGDA, Irgacure 819	Cell behaviors and neuronal differentiation were assessed in vitro	−	3D printed NGCs improved cell function	^[^ ^52^ ^]^
	Assembling spheroids for constructing NGCs	NHDF, silicone tube	5‐mm nerve gap in rat	8 weeks	3D NGCs promoted nerve regeneration	^[^ ^119^ ^]^
	Assembling spheroids for constructing NGCs	G‐MSCs, type I collagen gel	5‐mm nerve gap in rat	12 weeks	NGCs promised potential application for repair and regeneration of peripheral nerve defects	^[^ ^11l^ ^]^
Multiple lumen linear NGCs	DLP‐based rapid continuous 3D printing	PDMS, GelMA, PEGDA, LAP	4‐mm nerve gap in rat	11 weeks	Rats showed promising recovery of motor function and sensation	^[^^62c^^]^
	Micro‐MRI technique, a single nozzle melt 3D printer	−	−	−	This method could provide a template for the design of downstream nerve graft model	^[^ ^1a^ ^]^
	Mandrel adhesion method	PCL, porous collagen‐based beads (CultiSphers)	Cell behaviors were assessed in vitro	−	3D printed NGCs improved cell function	^[^ ^63^ ^]^
Bifurcated 3D printed NGCs	Layer‐by‐layer 3D printing	Polyethylene‐like material	3 ‐mm sciatic nerve gap before trifurcation in rat	12 weeks	3D printed NGCs with interposed autograft could prevent neuroma formation	^[^ ^72^ ^]^
	An imaging‐coupled 3D printing methodology	Silicone, NGF, GDNF, gelatin methacrylate hydrogel	10 ‐mm complex nerve gap in rat	3 months	The platform had a significant impact on both the fundamental understanding of complex nerve injuries	^[^^27a^^]^
Multichannel NGCs and bifurcating NGCs	Layer‐by‐layer fabrication procedure	Sodium hyaluronate, I2959, HAbp, HA, SCs	Cell behaviors were assessed in vitro	−	3D printed NGCs improved cell function	^[^ ^66^ ^]^
Single lumen NGCs, multichannel NGCs, and bifurcating NGCs	3D printed molds	CryoGelMA gel, A‐MSC	10 ‐mm sciatic nerve gap in rat	16 weeks	NGCs supported the re‐innervation across 10 ‐mm sciatic nerve gaps	^[^ ^129a^ ^]^
Irregular 3D printed NGCs	A microfluidic approach, extrusion‐based bioprinting	Gelatin, MA, chitosan, I2959	Cell behaviors were assessed in vitro	−	3D printed NGCs improved cell function	^[^ ^75^ ^]^
	EHD‐jet 3D printing	PCL, PAA	Cell behaviors were assessed in vitro	−	3D printed NGCs improved cell function	^[^ ^76^ ^]^
	An extrusion‐based type of 3D printing	PDL, RGD, PHH	Cell behaviors were assessed in vitro	−	3D printed NGCs improved cell function	^[^ ^77^ ^]^
	EHD‐jetting 3D printing	PCL, glacial acetic acid	Cell behaviors were assessed in vitro	−	3D printed NGCs improved cell function	^[^ ^19^ ^]^
	Layer‐by‐layer fabrication procedure	SCs, alginate, HA, fibrinogen, thrombin TISSEEL VHSD kits	Cell behaviors were assessed in vitro	−	3D printed NGCs directed the extension of dorsal root ganglion neurites	^[^ ^78^ ^]^
	Extrusion‐based bioprinting	Gelatin/alginate hydrogel, SCs	Cell behaviors were assessed in vitro	4 weeks	NGCs improved cell adhesion and related factor expression	^[^ ^87^ ^]^
Multifunctional 3D printed NGCs	A novel electrohydrodynamic jet 3D printing	PCL, PPy	Cell behaviors were assessed in vitro	−	PPy‐based conductive scaffolds had the potential for peripheral neuronal regeneration	^[^ ^91^ ^]^
	DLP	GelMA hydrogels, MPEG‐PCL nanoparticles, LAP, SCs, HUVECs	5‐mm sciatic nerve gap in rat	3 months	NGCs induced the recovery of sciatic nerve injuries in vivo	^[^ ^93^ ^]^
	Fused deposition modeling 3D printing	PC fiber, PLO, DWCNTs, NSCs	Cell behaviors were assessed in vitro	−	3D printed NGCs improved cell function	^[^ ^96^ ^]^
	EHD‐jet 3D printing	rGO, PCL, PC12 cells	Cell behaviors were assessed in vitro	−	NGCs could support the differentiation of PC12 cells.	^[^ ^101^ ^]^
	Layer‐by‐layer casting	Graphene, PCL, PDA, RGD	15‐mm nerve gap in rat	18 weeks	NGCs promoted successful axonal regrowth and remyelination	^[^ ^28b^ ^]^
	Stereolithography	PCL, NGF, camphorquinone, ethyl 4‐dimethyl aminobenzoate	15 ‐mm critical size sciatic nerve defect in rat	16 weeks	3D printed NGCs could lead to a better functional regenerative outcome	^[^^62b^^]^
	DLP based continuous 3D printing process	Collagenase I, GelMA, LAP, SCs, PC12 cells	10‐mm sciatic nerve gap in rat	3 months	NGCs could efficiently repair the injured nerves	^[^ ^118^ ^]^

Abbreviations: 3D, three‐dimensional; A‐MSC, adipose‐derived mesenchymal stem cell; CryoGelMA, cryopolymerized gelatin methacryloyl; dECM, decellularized extracellular matrix; DLP, digital light processing; DWCNT, double‐walled carbon nanotube; EHD, electrohydrodynamic; EHS, Engelbreth‐Holm‐Swarm; GDNF, glial cell line‐derived neurotrophic factor; GelMA, gelatin methacryloyl; G‐MSC, gingiva‐derived mesenchymal stem cell; HA, hyaluronic acid; HAbp, HA‐binding protein; HUVEC, human umbilical vein endothelial cell; I2959, irgacure 2959; LAP, lithium phenyl‐2,4,6‐trimethyl‐benzoylphosphinate; MA, methacrylic anhydride; micro‐MRI, micro‐magnetic resonance imaging; NGC, nerve guide conduit; NGF, nerve growth factor; NHDF, normal human dermal fibroblast; NIH‐3T3 cell, mouse embryonic cell; NSC, neural stem cell; PAA, poly(acrylic acid); PC, polycarbonate; PCL, poly(ε‐caprolactone); PDA, polydopamine; PDL, poly(D‐lysine); PDMS, polydimethylsiloxane; PEG, poly(ethylene glycol); PEGDA, poly(ethylene glycol) diacrylate; PHH, PHEMA hydrogel; PLO, poly(ʟ‐ornithine); PPy, polypyrrole; RGD, arginine‐glycine‐aspartic acid; rGO, reduced graphene oxide; SC, Schwann cell.

### 2.1 Single Lumen Linear 3D Printed Nerve Guide Conduits

**Figure 2:**
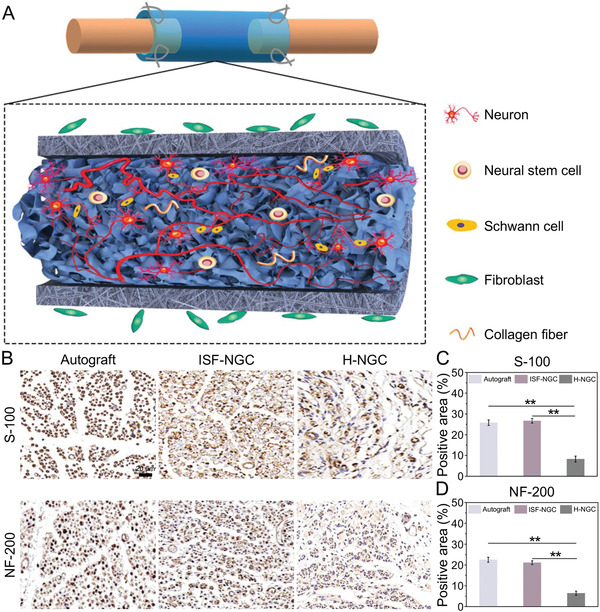
Characteristics of single lumen linear 3D printed NGC. A) Speculative scheme of ISF‐NGC in enhancement of nerve regeneration. B) Immunohistochemical analysis is of the regenerated nerves eight weeks postoperative. Scar bars, 20 µm. Statistical analysis of regenerated nerve by calculating. C) Percentage of S‐100 positive area and D) Percentage of NF‐200 positive area. Reproduced with permission.^[^ ^40^ ^]^ Copyright 2019, Wiley‐VCH.

Traditional NGCs are simple hollow tubular structures because it is difficult and time‐consuming to manufacture NGCs with complex structures.^[^ ^11^, ^27^, ^36^ ^]^ They are usually made of natural or synthetic polymers.^[^ ^37^ ^]^ The first attempt of hollow NGC in nerve repair was in 1881. In a dog model, a hollow bone was used to bridge the nerve defect but was unsatisfactory.^[^ ^11a^ ^]^ In 1983, Williams et al. used neural stem cells (NSCs) and an impermeable hollow silicon NGC to bridge a 10‐mm rat sciatic nerve defect and studied the process of PNR. The axons reached the distal stump after three weeks, and they studied the cellular and molecular mechanisms of PNR.^[^ ^38^ ^]^ After decades of intensive research, a deeper understanding of the mechanism of PNR has gradually emerged.^[^ ^39^ ^]^ At present, all evidences suggest that adding luminal filler to NGC can enhance the effect of PNR (**Figure** **2**).^[^ ^40^ ^]^ Nevertheless, single lumen linear hollow NGCs, approved by the Food and Drug Administration (FDA), are still the most commonly used NGCs in clinical practice because of their practicality.^[^ ^41^ ^]^

3D printing can prepare macroporous NGCs, which are essential for exchanging nutrients and oxygen (O_2_) and the proliferation of endothelial cells.^[^ ^28b^ ^]^ 3D printing can also control the pore size and distribution in the material matrix.^[^ ^42^ ^]^ Zhang et al. used computational design to study the different NGC structural features and finally designed an ideal NGC with suitable performance.^[^ ^43^ ^]^ NGC performances, including porosities, specific surface areas, mechanical properties, permeabilities, and so forth, were also revealed.^[^ ^44^ ^]^ In particular, some studies have reported that single lumen linear hollow NGCs could reduce neuroma and scar formation, ineffective axonal extension, and fibroblast infiltration.^[^ ^7^, ^45^ ^]^

In addition to the structure and performance of NGC, the diameter of conduit is an essential factor affecting PNR. In a previous study, NGCs with a diameter of 200−300 µm were effective in repairing 1‐cm long sciatic nerve defect.^[^ ^46^ ^]^ Similar axonal growth was observed in NGCs with a diameter of 150−300 µm.^[^ ^47^ ^]^ However, Krych et al. found that NGCs that were larger than 450 µm in diameter caused neurodevelopmental dysplasia in the rat spinal cord, with a decrease in the number of axons regenerated in NGCs two months after implantation.^[^ ^48^ ^]^ This phenomenon may be caused by local inflammation, ineffective axonal growth, and scar invasion into the NGC.^[^ ^48^ ^]^ In an experiment with astrocytes cultured in vitro, the small diameter (180 µm) conduit enhanced astrocyte alignment by altering their proliferation morphologies.^[^ ^49^ ^]^ Thus, an oversized conduit may result in misdirected axonal regeneration, while an undersized one may result in nerve entrapment.^[^ ^11^, ^50^ ^]^ These studies suggest that the sizes of NGCs have a significant effect on PNR, but more detailed research is needed in the future.

Unfortunately, single lumen linear hollow NGCs are not effective for repairing nerve defects. The low permeability of conduit wall to GFs and NTFs can hinder the process of nerve regeneration to some extent.^[^ ^51^ ^]^ In addition, hollow NGCs sometimes lack the physical, chemical, and biological cues that promote nerve growth. Even worse, linear hollow NGCs can lead to misallocation of axons or incomplete regeneration of nerves because axonal fibers of the same neuron are dispersed to different target tissues, which are sometimes innervated by multiple nerves.^[^ ^52^ ^]^ This may be a reason for the poorer functional recovery of linear hollow NGCs compared to autografts.^[^ ^53^ ^]^ For studies in animal models, this type of NGCs can repair nerve defects, which are less than 30 mm.^[^ ^11^, ^54^ ^]^

Researchers have tried various methods to improve the performances of hollow NGCs, thereby improving axon growth in long‐distance nerve defects.^[^ ^55^ ^]^ The internal microstructures and mechanical properties of NGCs may be determinants in promoting axonal growth. Yoo et al. combined 3D printed collagen hydrogel and electrospun poly(L‐lactide‐*co*‐ε‐caprolactone) (PLCL) membrane to prepare a single lumen NGC to repair PNI. The results showed that NGCs had significant promoting effects on axonal growth, myelin regeneration, and nerve function recovery, which provided a valuable reference for developing single lumen linear NGC.^[^ ^56^ ^]^ Zhang et al. designed a self‐adhesive bandage consisting of two layers of hydrogel that can incorporate nanomedicine to form an envelope around an injured nerve to promote nerve regeneration. The experimental results showed that the bandage can promote the repair of injured nerves, indicating its potential application in nerve regeneration.^[^ ^57^ ^]^ Overall, the single lumen linear hollow conduits are the initial design version of NGCs.^[^ ^58^ ^]^ Although hollow NGCs cannot fully meet clinical requirements, they are more clinically acceptable than other types of NGCs.^[^ ^59^ ^]^

### 2.2 Multi‐Lumen Linear 3D Printed Nerve Guide Conduits

Recent studies have shown that multi‐lumen NGCs are superior to single lumen NGCs in promoting PNR.^[^ ^60^ ^]^ This can be explained by 1) the longitudinally aligned lumen in the conduit can act as a microtubule with a large surface area required for the synthesis of the basement membrane, which has a significant positive effect on the attachment, proliferation, and migration of SCs; 2) multiple longitudinal lumens within NGCs reduce axon dispersion and promote the longitudinal extension of axons; 3) multiple lumens arranged longitudinally can reduce the misorientation rate of regenerating axons connecting to distal nerve stumps.^[^ ^36^, ^61^ ^]^ Therefore, it is believed that multi‐lumen NGCs are more suitable than single lumen NGCs for promoting PNR.^[^ ^62^ ^]^ Moore et al. were among the first researchers to report multi‐lumen NGCs. In 2006, they prepared NGCs with multiple lumens by injection molding using a rapidly evaporating solvent.^[^ ^62d^ ^]^ The large specific surface area in multi‐lumen conduits promotes cell adhesion and provides suitable directional guidance for axonal growth.^[^ ^63^ ^]^

**Figure 3:**
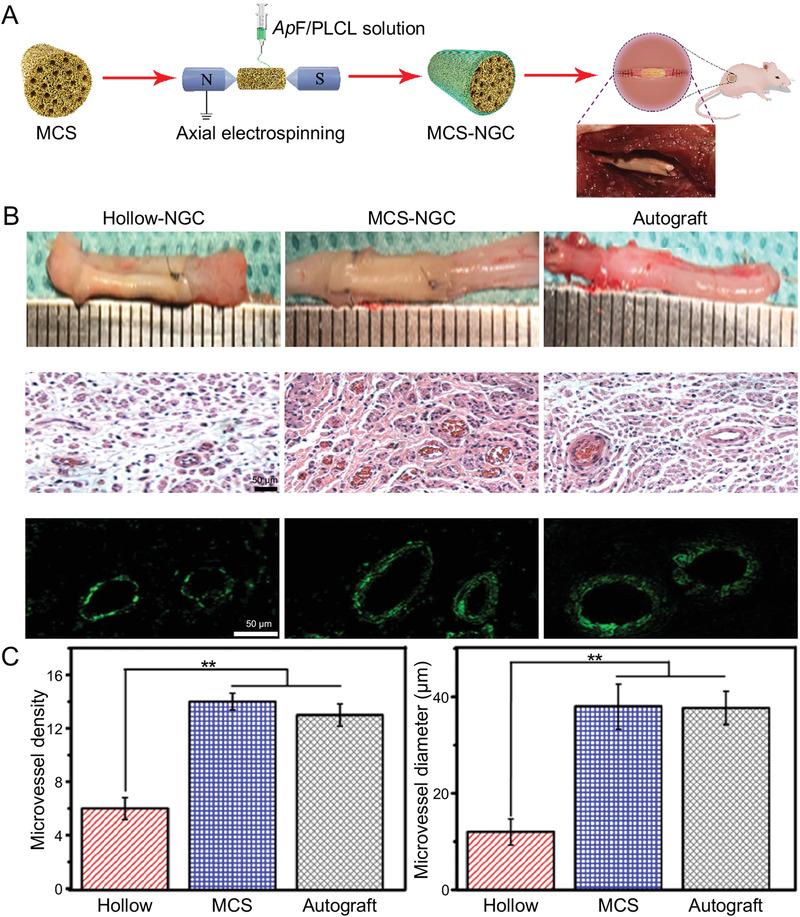
Fabrication and implantation of MCS‐NGC for sciatic nerve repair. A) Schematic illustration of fabrication of MCS‐NGC and photograph of implanted nerve conduits. MCS, multichannel sponge. B) General observation of regenerated nerve tissue and angiogenesis evaluation in sciatic nerve regeneration at 12 weeks postoperatively. C) Density and diameter of newly formed microvessels based on H&E‐stained sections. H&E, hematoxylin and eosin. Reproduced with permission.^[^ ^65^ ^]^ Copyright 2020, Elsevier Ltd.

Results from a series of in vitro or in vivo experiments have demonstrated that 3D printed multi‐lumen NGCs have great potential to repair PNI. Owens et al. used several biomaterials, SCs, and mouse bone marrow‐derived mesenchymal stem cells (BM‐MSCs) to prepare multi‐lumen linear NGCs. Three weeks after conduit implantation in the sciatic nerve injury rat model, electrophysiological examination showed that motor and sensory functions were restored.^[^ ^64^ ^]^ Yao et al. reported that multi‐lumen linear NGCs could promote the attachment of SCs, reduce the dispersion of regenerated axons, and effectively promote the recovery of nerve function.^[^ ^36^ ^]^ Wang et al. prepared multi‐lumen nerve conduit using *Antheraea pernyi* silk fibroin/poly(L‐lactic acid‐*co*‐ε‐caprolactone)/graphene oxide (*Ap*F/PLCL/GO) nanofiber (**Figure** **3**). The conduit had multiple parallel lumens, a suitable degradation rate, and improved mechanical properties.^[^ ^65^ ^]^ Suri et al. prepared multi‐lumen linear NGC using 3D printing technology. Calcein staining showed that SCs inoculated in the conduit remained viable after 24 h.^[^ ^66^ ^]^

Conventional preparation methods cannot adjust the structural parameters of NGCs, such as the inner diameter and wall thickness, which can affect the efficacy of repairing nerves.^[^ ^11^, ^67^ ^]^ In addition, the preparation process should ideally incorporate the patient's imaging data so that personalized treatment can be provided to the patient.^[^ ^66^ ^]^ The digital micromirror array device manufacturing system has received much attention for its ability to prepare NGCs that mimic the structure of natural nerve tissue. Lee et al. conducted an in vivo study using functional tripeptide sequence multi‐lumen NGCs in a 10‐mm sciatic nerve transection rat model. The outcome showed significantly higher electrophysiological activity in the NGC group compared to the autograft group after eight weeks.^[^ ^68^ ^]^ The use of personalized 3D printing technology to prepare NGCs with advanced structures and controllable parameters will effectively promote the clinical application of NGCs in the future.

However, some studies have shown that multi‐lumen linear NGCs sometimes interfere with axonal regeneration, thus affecting the efficacy of nerve repair.^[^ ^69^ ^]^ Stereolithography was used to prepare single and multi‐lumen poly(ethylene glycol) (PEG) NGCs to repair 10‐mm sciatic nerve defects in rats. Nerve regeneration was observed in 70% of the animals implanted with single lumen NGC, while axonal regeneration was poor in the multi‐lumen NGC group.^[^ ^69^ ^]^ It has been hypothesized that one of the reasons for inefficient nerve regeneration in the multi‐lumen NGC group is the slow degradation of NGC.^[^ ^62b^ ^]^ From the current findings of multi‐lumen linear NGCs, it seems that these NGCs cannot provide sufficient space for axon growth.^[^ ^70^ ^]^ Therefore, lumen diameter, material selection, and surface topography are critical factors in designing multi‐lumen linear NGC structures.^[^ ^69^ ^]^ More studies are needed to explore this issue, as it is unclear how multi‐lumen linear NGCs affect the PNR process.

### 2.3 Bifurcated 3D Printed Nerve Guide Conduits

**Figure 4:**
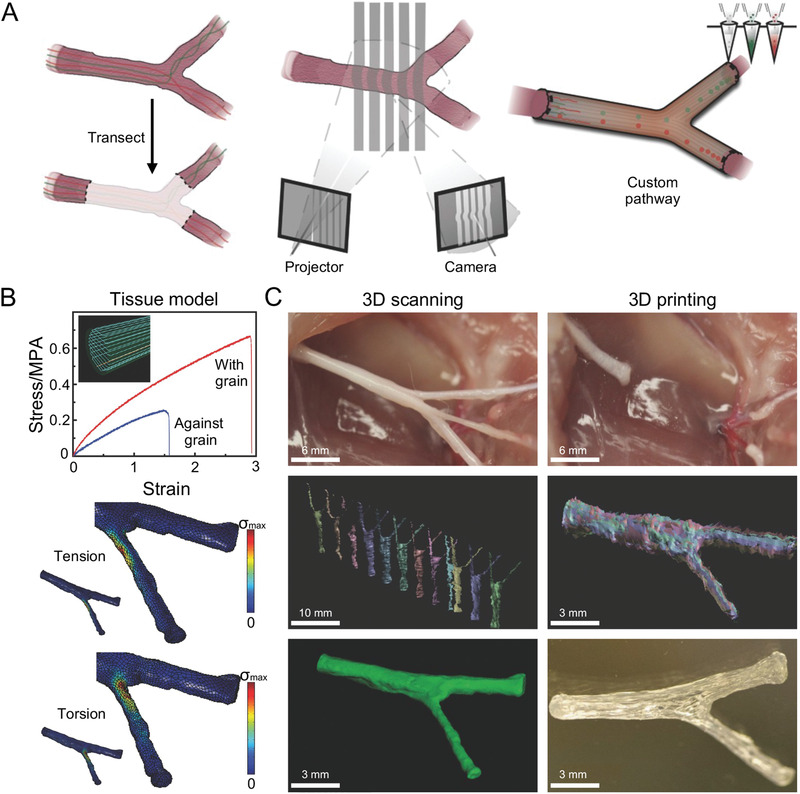
Personalized nerve regeneration pathways of bifurcated 3D printed NGC. A) Personalized nerve regeneration pathways enabled by 3D scanning and printing. B) Mechanical characterization and computational analysis of pathways. C) 3D printed complex nerve pathways from 3D scanned bifurcating nerves. Reproduced with permission.^[^ ^27a^ ^]^ Copyright 2015, Wiley‐VCH.

In addition to cylinders, peripheral nerves can also be bifurcate and taper. Conventional NGC preparations are usually performed around cylindrical substrates, so the products are limited to linear structures.^[^ ^27a^ ^]^ The prepared NGCs with nerve structures and biological functions using 3D printing technology has a promising future.^[^ ^27a^ ^]^ Johnson et al. developed a 3D printing strategy for producing complex structural NGCs using silicone as the raw material (**Figure** **4**A,B).^[^ ^27a^ ^]^ The method includes the following critical steps: 1) identifying the target nerve; 2) imaging the target nerve structure and producing the corresponding 3D model; 3) preparing a 3D printed NGC with physical and biochemical cues. This method provides a new idea for regenerating the bifurcation nerve, which is difficult to achieve with conventional NGCs.^[^ ^27a^ ^]^

Johnson et al. chose the bifurcation of rat sciatic nerve as a model and then used 3D structured light scanning technology to image and prepare the nerve model (Figure 4C).^[^ ^27a^ ^]^ This technique was combined with tissue imaging to generate a scanned sample of nerve finally.^[^ ^27a^ ^]^ They used the scanned sample as a model for 3D printed pathway intelligence to directly prepare personalized precise nerve conduits to facilitate regeneration of complex nerve defects.^[^ ^27a^ ^]^ Thus, this study suggests that 3D printing may provide a method to regenerate complex neural structures and open the way for individualized treatment for various nerve injuries.^[^ ^27a^ ^]^ With increased fabrication resolution, it is convenient to customize implants for patients using imaging data, which is one of the advantages of 3D printing.^[^ ^71^ ^]^ Indeed, nerve tissue imaging data have been used to guide the preparation of bifurcated structural NGCs. With the increased resolution of 3D printing, it is possible to go to the level of nerve bundles for nerve repair, thus allowing the repair of nerve defects over longer distances.

The bifurcated Y‐shaped nerve conduit also has an essential role in inhibiting neuroma formation. Bolleboom et al. used 3D printed Y‐shaped conduit to prevent the development of neuroma.^[^ ^72^ ^]^ Using a combination of Y‐shaped NGC and nerve autograft, they produced a closed loop that induced axonal regeneration into the Y‐shaped conduit. The results demonstrated that the irregular arrangement of regenerated axons reduced neuroma formation.^[^ ^72^ ^]^ This strategy is suitable for single nerve injuries, and the procedure is relatively simple. In addition, if the Y‐shaped conduits are made of biodegradable materials, it can also prevent nerve entrapment.^[^ ^72^ ^]^ 3D printed Y‐shaped NGCs have potential applications in nerve repair and prevention of neuroma formation. It is a promising technology, which has the potential to prepare Y‐shaped bifurcated NGCs.

To date, no tissue engineering NGC can fully mimic the structure and composition of healthy nerve. In addition to guiding axonal regeneration and providing mechanical support and protection, the NGC should also differentiate between motor and sensory nerve bundles to guide different nerve bundles bridging to different target organs.^[^ ^73^ ^]^ To achieve this nerve regeneration effect, it may be necessary to correspond the internal structure of NGC to the corresponding nerve bundle in the injured nerve separately so that sensory‐specific SCs surround sensory axons and motor‐specific SCs surround motor axons.^[^ ^74^ ^]^ Thus, the favored nerve graft substitute is ultimately composed of the appropriate extracellular matrix (ECM) protein, phenotype‐specific supporting cells, vascular system, and mechanical integrity. All of these are perfectly integrated with the proximal and distal nerve stumps.

### 2.4 Irregular 3D Printed Nerve Guide Conduits

Nerve tissue is a complex 3D structure composed of multiple interacting cells.^[^ ^75^ ^]^ Assessment of nerve injury is not easy, especially when various nerves are injured.^[^ ^76^ ^]^ The different nerve injury conditions of patients have encouraged researchers to develop more individualized treatment methods.^[^ ^77^ ^]^ In this case, simple structures made of NGC using conventional fabrication methods are not sufficient to solve the current problem, as more non‐tubular structures are needed to simulate the normal physiological structure of injured nerve.^[^ ^78^ ^]^

**Figure 5:**
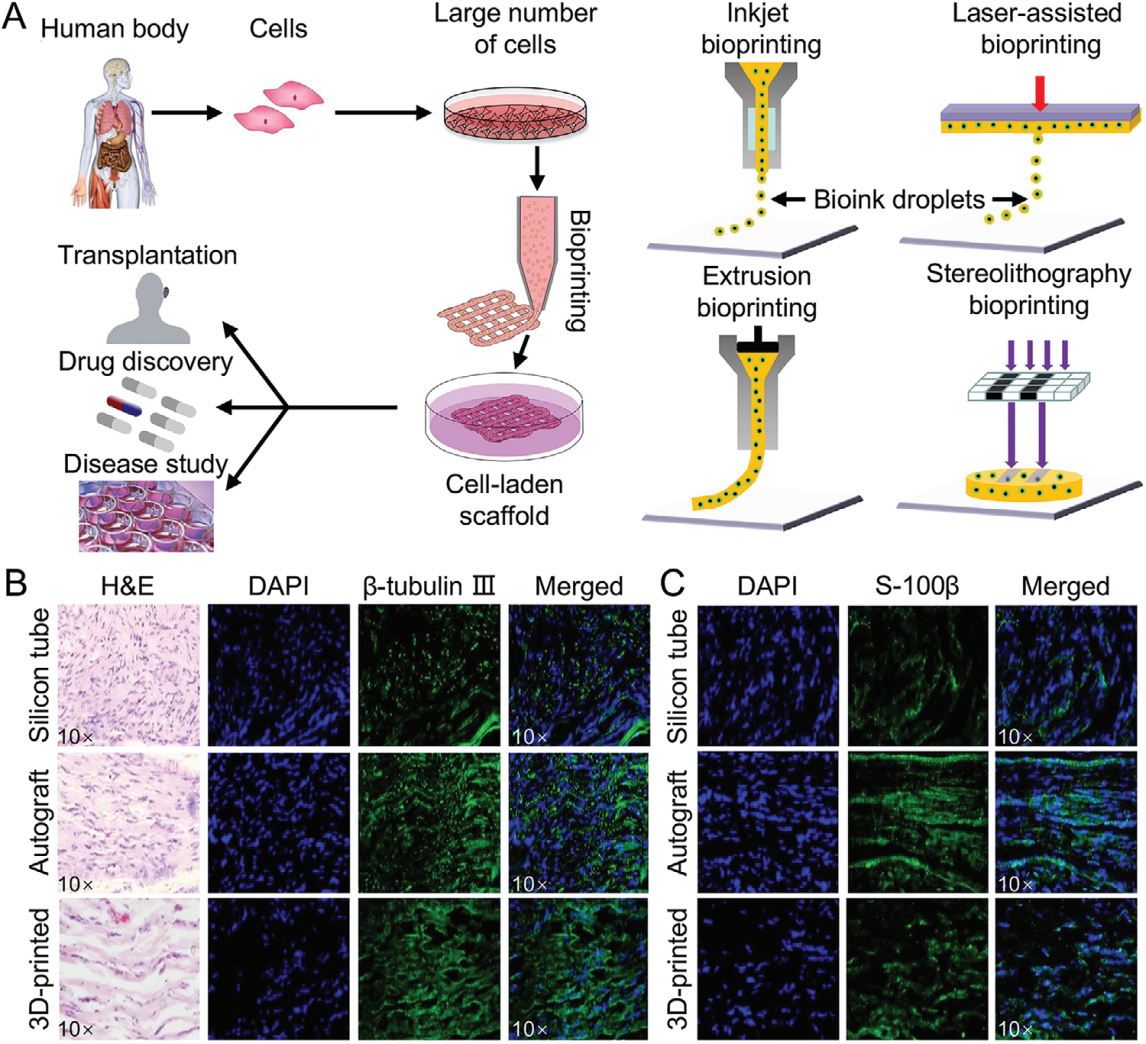
Production principle and application of irregular 3D printed NGC. A) Production principle of irregular 3D printed NGC. B) Histological analysis of newly regenerated rat facial nerves. C) Immunohistochemistry showed increased expression of S‐100*β* in regenerated facial nerve from group with 3D bio‐printed construct transplantation as compared with silicon tube control group. A) Reproduced with permission.^[^ ^80^ ^]^ Copyright 2016, Elsevier Ltd. B,C) Reproduced with permission.^[^ ^81^ ^]^ Copyright 2018, Springer Nature.

Recently, the combination of 3D printing technology and stem cell therapy has attracted more attention.^[^ ^79^ ^]^ In neural tissue engineering, 3D bioprinting is considered a promising technology to develop cell‐loaded neural scaffolds (**Figure** **5**A).^[^ ^80^ ^]^ For example, Owens et al. used a bioprinter to prepare neural grafts loaded with BM‐MSCs and SCs.^[^ ^64^ ^]^ Similarly, Zhang et al. prepared a 3D bioprinted nerve scaffold constructed from human gingival‐derived mesenchymal stem cells (G‐MSCs). They incubated G‐MSCs for 14 days and then transplanted them into a facial nerve defect rat model. After 12 weeks, the cell‐loaded scaffold repaired 5‐mm facial nerve buccal branch defect in rat similarly to nerve autograft (Figure 5B,C).^[^ ^81^ ^]^ Thus, 3D bioprinting is a promising technology that can be used for structural design and bioprocessing of personalized irregular NGCs.^[^ ^27^, ^82^ ^]^

Nerve tissue engineering combines biomaterials with a variety of cells and bioactive molecules to provide an optimal extracellular microenvironment for nerve regeneration.^[^ ^83^ ^]^ Human‐derived cells, including human‐derived endothelial cells, fibroblasts, mesenchymal cells, and so forth, can be used in these 3D scaffolds, showing great potential for application.^[^ ^84^ ^]^ Adipose‐derived mesenchymal stem cells (A‐MSCs) and BM‐MSCs transplanted into nerve scaffolds can differentiate into Schwann‐like cells that support axonal renewal. Considering the inevitable cell leakage and migration, irregular scaffolds can carry cells better than linear NGCs as long as the 3D bioprinted irregular scaffolds do not collapse. Some studies reported that various GFs were added to NGCs by direct injection into the lumen or additional transport systems to promote PNR.^[^ ^62^, ^85^ ^]^ However, the effectiveness of irregular scaffolds for nerve repair is inevitably reduced by cell leakage and short‐term activity. However, if the scaffold is made of cells expressing GFs, this problem can be effectively solved.^[^ ^86^ ^]^

However, when repairing long‐distance nerve defects, 3D bioprinted irregular scaffolds usually perform poorly in vivo compared to autografts. Irregular scaffolds should avoid additional pressure on surrounding tissues and not cause compression of regenerating axons. Therefore, the mechanical property, stability, and suture‐ability of biomaterials should be considered during irregular neural scaffold preparation. However, most bioinks simply mix hydrogels and cells, limiting the positioning and proliferation of various cells in 3D bioprinted irregular scaffolds.^[^ ^87^ ^]^

**Figure 6:**
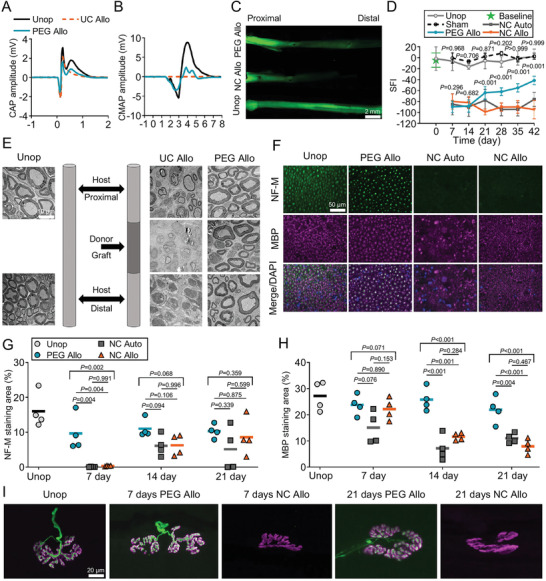
Neural repair effects of 3D printed NGC. A) Representative compound action potential (CAP) recordings from unoperated control sciatic nerve, PEG‐fused PNA, and negative control PNA. B) Representative compound muscle action potential (CMAP) recordings after stimulating sciatic nerves, recorded from tibialis anterior (TA) muscle. C) Intra‐axonal diffusion evidence for immediate restoration of axonal continuity in PEG‐fused PNA. D) SFI scores show functional recovery over time. E) TEM images showing axons and myelin in cross sections of unoperated control nerve (left) and proximal, graft, and distal segments of negative control PNA (middle) and PEG‐fused PNA (right) at 21 days PO. TEM, transmission electron microscopy. F) Fluorescence images showing cross sections of unoperated control nerve (Unop), PEG‐fused PNA (PEG Allo), NC autograft (NC Auto), and NC PNA (NC Allo) at 7 days PO. G) Time course of % NF‐M staining areas from 7 to 21 days PO. H) Time course of % MBP staining areas from 7 to 21 days PO. I) Confocal images of NMJs immunostained for NF‐M (green) and acetylcholine receptor (AchR, magenta) in animals of unoperated control, PEG‐fused, and negative control PNAs groups at 7^th^ and 21^st^ day PO. Reproduced with permission.^[^ ^89^ ^]^ Copyright 2020, Wiley‐VCH.

Many problems may be encountered when preparing nerve scaffolds using biomaterials, including limited binding of cells within the nerve scaffold and foreign‐body reactions caused by the scaffold, and so on.^[^ ^88^ ^]^ Eliminating foreign‐body responses to scaffolds can reduce the risk of infection to some extent. Smith et al. successfully fused PEG to peripheral nerve allograft (PNA) of the sciatic nerve and examined the innate and adaptive immunity of this PEG‐fused PNA by comparative morphology, immunohistochemistry, and gene exposure characteristics generally associated with acute cellular rejection (**Figure** **6**).^[^ ^89^ ^]^ The results suggested that the PEG‐fused PNA was not acutely rejected in remote donor/host without tissue‐matched or immunosuppressive drug treatment. In short, the PEG‐fused PNA well illustrated the immune tolerance of allogeneic tissues surviving in a non‐immune privileged microenvironment and could significantly improve the outcome of PNI compared to current protocols. Therefore, exploring suitable printed biomaterials is essential for producing 3D printed biological conduits.^[^ ^90^ ^]^

## 3 Multifunctional 3D Printed Nerve Guide Conduits

Recent development in multifunctional NGCs has changed the concept of traditional nerve conduits.^[^ ^91^ ^]^ They can actively promote axonal regeneration and nerve growth through various methods.^[^ ^92^ ^]^ These methods can be used individually or in combination, such as chemical cues (chemical structures), physical cues (topographic guidance or electrical stimulation), and biological cues (cells or factors).^[^ ^93^ ^]^ A variety of preparation techniques have been used to prepare multifunctional NGCs, such as electrospinning, mold‐molding, and dip‐coating.^[^ ^94^ ^]^ Vashisth et al. used the electrospinning technique to prepare NGC, making it safe and effective to deliver quercetin to nerve defect, so enhanced the viability of SHY5Y neuronal cells.^[^ ^95^ ^]^ However, these techniques can only prepare the limited precise structures. In contrast, 3D printing allows preparing implants that closely match the nerve defects.^[^ ^96^ ^]^ In addition, biological cues, such as bacteria,^[^ ^97^ ^]^ SCs,^[^ ^98^ ^]^ stem cells,^[^ ^99^ ^]^ and NTFs,^[^ ^100^ ^]^ can be co‐printed with biomaterials into multifunctional NGCs and promote axon elongation and myelination.

**Figure 7:**
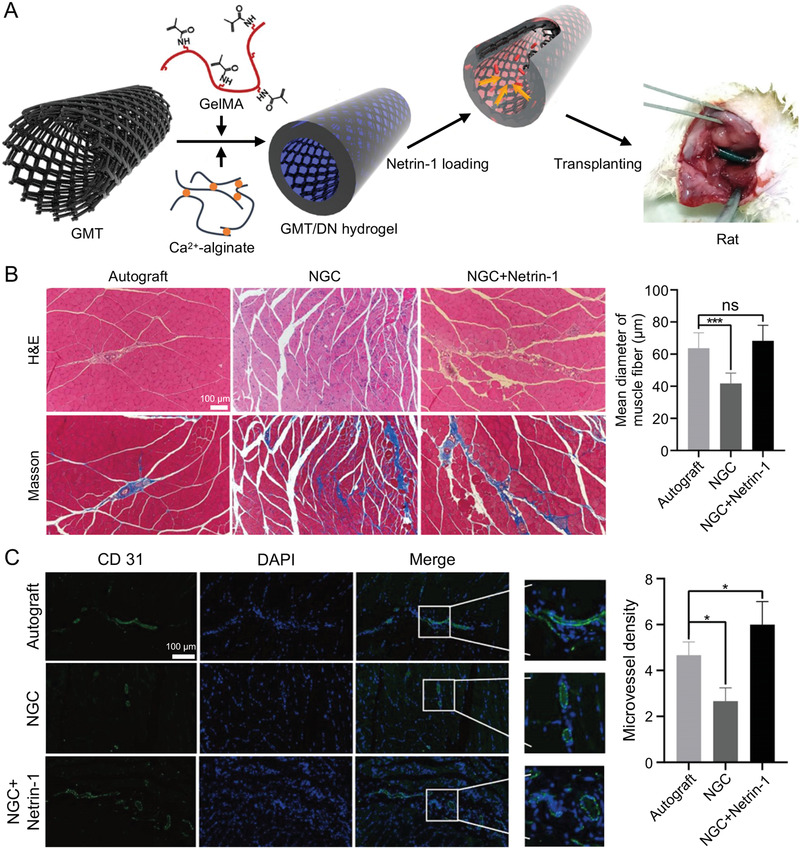
Effectiveness of netrin‐1‐loaded GMT/hydrogel NGC repair of peripheral nerves. A) Schematic illustration of netrin‐1‐loaded GMT/hydrogel NGC. B) H&E and Masson staining of gastrocnemius muscles and mean diameter of muscle fibers. C) Immunofluorescence staining of CD31 and microvessel density by CD31 staining. Reproduced with permission.^[^ ^103^ ^]^ Copyright 2021, American Chemical Society.

A variety of materials have been used to produce multifunctional NGCs, while stronger mechanical materials ensure the structural stability of NGCs, and softer materials provide better biocompatibility.^[^ ^101^ ^]^ In a previous study, Gao et al. demonstrated that sodium alginate could be functionalized to promote proliferation and differentiation of endothelial progenitor cells while maintaining the suitability for 3D coaxial printing.^[^ ^102^ ^]^ Huang et al. fabricated a 3D graphene mesh tube (GMT) using nickel mesh as a template (**Figure** **7**).^[^ ^103^ ^]^

**Figure 8:**
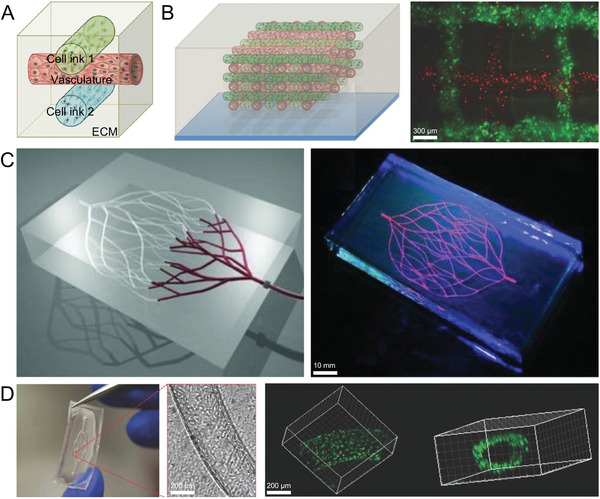
3D printed multifunctional permutable vascularized tissue constructs. A) Schematic views of our 3D bioprinting approach. B) Schematic view and fluorescence image of an engineered tissue construct cultured. C) 3D microvascular networks within a hydrogel reservoir. D) Optical image of representative microchannel within a 2D vascular network. A,B,D)Reproduced with permission.^[^ ^107c^ ^]^ Copyright 2014, Wiley‐VCH. C) Reproduced with permission.^[^ ^104^ ^]^ Copyright 2011, Wiley‐VCH.

PNI may sometimes affect perineural tissues, and reestablishing blood flow to perineural tissues may improve nerve regeneration.^[^ ^104^ ^]^ NGC is anastomosed to the vessels in the nerve defect area to ensure proper blood circulation.^[^ ^105^ ^]^ Initial attempts to construct vascularized NGCs consisted of inserting a sural vessel or subcutaneous artery into a silicone conduit and implanting it into the sciatic nerve defect in rats.^[^ ^106^ ^]^ Arterial blood supply significantly increased the number, diameter, and conductivity of neurons in the early post‐implantation period compared to the conventional NGC experimental group. Mitsuzawa et al. reported NGCs composed of human induced pluripotent stem cells‐derived mesenchymal stem cells (iPSC‐MSC) in a rat model of nerve defect. The results indicated that the transplanted iPSC‐MSCs promoted vascular regeneration in areas of PNR. Researchers are currently exploring the bioprinting of entire organ or tissue structures embedded in blood vessels formed by the directed arrangement of endothelial cells, which makes it possible to prepare NGCs containing blood vessels (**Figure** **8**).^[^ ^13^, ^107^ ^]^

Electrical signals have been shown to promote nerve regeneration and functional recovery.^[^ ^108^ ^]^ Studies demonstrated that electrical stimulation accelerated the healing of sciatic and facial nerve function in rodents.^[^ ^109^ ^]^ Both AC and DC electric fields promote the growth of dorsal root ganglion neurons and the release of NTFs from SCs.^[^ ^110^ ^]^ Experiments on patients with carpal tunnel syndrome have demonstrated that electrical stimulation can reduce pain and accelerate axonal regeneration.^[^ ^111^ ^]^ Short‐term electrical stimulation over multiple days has been reported to improve the prognosis of patients, suggesting that electrical stimulation is essential for the recovery of neurological disease.^[^ ^112^ ^]^

**Figure 9:**
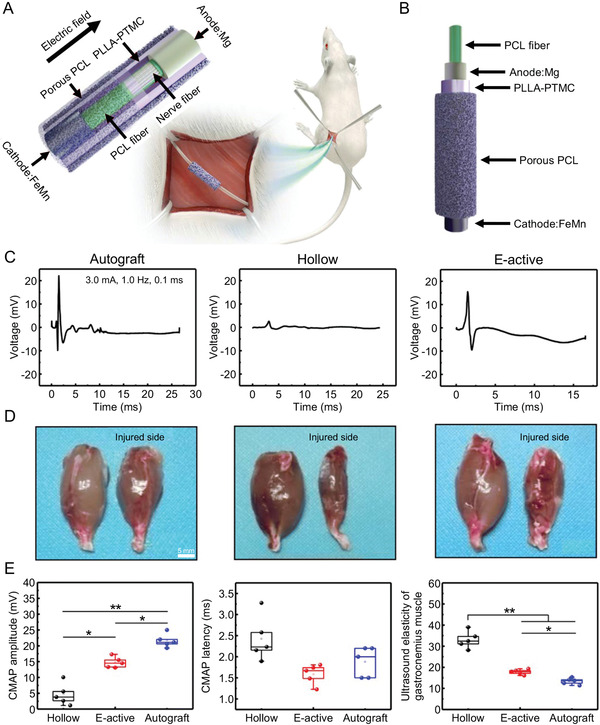
A biodegradable, self‐electrified, and miniaturized conduit device for neuroregenerative medicine. A) Schematic illustration of device for sciatic nerve regeneration. B) Schematic exploded illustration of device. C) Representative CMAP at injured side. D) Gross images of isolated gastrocnemius muscles. E) Statistical analysis of CMAP amplitude at injured side, statistical analysis of CMAP latency at injured side, and statistical analysis of ultrasound elasticity of gastrocnemius muscles from injured limb. Reproduced with permission.^[^ ^114^ ^]^ Copyright 2020, American Association for the Advancement of Science.

Some NGCs can use wirelessly powered electrodes to generate electrical currents to promote sciatic nerve repair.^[^ ^113^ ^]^ However, further optimization of materials and preparation methods facilitates the development of wireless implantable NGCs, as the ideal devices require miniaturization, simplified manufacturing processes, and all biodegradable components to function without external device power. Wang et al. reported a biodegradable, self‐charging, and ultra‐miniaturized NGC for PNR facilitation, as shown in **Figure** **9**.^[^ ^114^ ^]^ The device consists of a primary cell made of magnesium (Mg) and iron‐manganese alloy (FeMn) electrodes on a biodegradable polymer conduit. It provided continuous electrical stimulation in nerve regeneration without additional surgical complications. The device can effectively promote sciatic nerve regeneration and functional recovery in rat. Conductive NGCs are increasingly used in the field of PNR. However, the use of conductive bioinks requires more in‐depth studies, including toxicity, safe dosage, biocompatibility, and so forth.^[^ ^115^ ^]^ For example, conductive silver nanoparticles are now widely used in various commercial products, but they can cause neurotoxicity, inflammation, and oxidative stress, ultimately reducing axonal growth.^[^ ^116^ ^]^

## 4 Conclusions and Perspectives

Currently, the recovery of sensory and motor functions of peripheral nerves after the injury is far from satisfactory. Only 3% of patients who undergo nerve repair surgery regain normal sensory function at five years, and less than 25% of patients regain normal motor function.^[^ ^117^ ^]^ For long‐distance (>3 cm) nerve defects, nerve autograft is still the standard modality for treatment. However, with the development of NGC research, it is expected to overcome some of the drawbacks of nerve autograft. Functionalized and conductive NGCs loaded with cells or factors, biodegradable gel filling, and nanopores may allow nutrient entry while limiting fibroblast infiltration and may promote rapid regeneration of the nerve defects. Various 3D bioprinting technologies have been applied to manufacture NGCs. The latest bioprinting trend is trying to simulate the neural tissue in living organisms as much as possible. Although such 3D bioprinted neural implants are still in their infancy, these studies provide a good foundation for further bioprinting of bionic nerve tissues.^[^ ^118^ ^]^

The main direction of future NGC research should aim at the multi‐functionalization of conduits through the integration of stem cells, nerve factors, and drugs.^[^ ^119^ ^]^ These cells, factors, or drugs can promote functional regeneration of nerve defects in longer distances. The use of stem cells holds new promise for PNR. iPSC have emerged as one of the most promising approaches in regenerative medicine. Combining iPSCs and their derived neurons with NGCs may be a future research direction. When manufacturing NGCs, special attention must be paid to the design of conduit, as the material and structure of conduit have essential implications for stem cell differentiation.^[^ ^120^ ^]^ The nerve factor is another central element of neural tissue engineering. It has two principal biological functions: Nourishing neuronal cells and promoting axonal growth. The preparation of neural conduits by loading nerve factors and using their ability to control the release of nerve factors to stimulate neural tissue regeneration is also a hot research topic in the future.

The inks used for 3D printing devices are classified as bioinks and decellularized inks. Bioinks loaded with cells, factors, and drugs must be prepared and processed in a suitable microenvironment for cell growth. In contrast, decellularized inks can be used for 3D printing in harsh microenvironments, such as extreme heat, intense ultraviolet light irradiation, and organic solutions. Since the elastic modulus of the human brain, spinal cord, and peripheral nerve tissues are 1, 10, and 100−500 kPa, respectively, better repair results may be obtained through using soft polymers with guaranteed mechanical strength.^[^ ^12^, ^121^ ^]^ Many different biomaterials have been developed to fabricate tissue engineering NGCs due to the susceptible nerve cells that require a suitable growth microenvironment and biologically guided cues, such as cells, factors, and drugs, to facilitate nerve tissue regeneration.^[^ ^28a^ ^]^ The ability of biological cues in 3D printed inks to survive and function over time remains demonstration. To achieve this goal, researchers have developed bioprinting materials that bind proteins to nerve ECMs, cells, or factors to improve the effectiveness of 3D printed products for promoting nerve regeneration.

Gene therapies have also been developed to treat PNI.^[^ ^122^ ^]^ Gene therapy eliminates toxic proteins at the site of injury, activates the germinal phenotype in injured nerves, and increases the expression of therapeutic signals in regenerating cells. Gene therapy increases cell communication between sensitive cells in a specific way and promotes the differentiation of stem cells.^[^ ^122^ ^]^ The regenerative properties of SCs decrease after denervation, and gene therapy can be targeted to this feature of SCs.^[^ ^123^ ^]^ Gene therapy often uses viral vectors to enhance the reproductive phenotype of SCs in NGCs.^[^ ^124^ ^]^ Various viral serotypes show different transcriptional spectrums and are better expressed in nerve cells.^[^ ^125^ ^]^ Gene therapy also targets the NTF gene and increases its expression, a factor that stimulates axonal regeneration.^[^ ^124^, ^126^ ^]^ However, a disadvantage of this therapeutic approach is that overexpression of NTF may have a negative feedback effect on axonal regeneration.^[^ ^124^, ^127^ ^]^ Such findings suggest that the approach requires further research to optimize the effect of nerve repair. Therefore, combining 3D printed NGC with gene therapy is also a promising approach for the treatment of PNI.

Unlike NGCs implanted in animals to repair nerves, in situ 3D printing technology serves as a transformative concept to directly repair PNI.^[^ ^12^ ^]^ Existing in situ 3D printing technique has been applied to repair cranial defect in rodent and skin wound in pig.^[^ ^128^ ^]^ Here, the in situ printer serves as a combination of scanner, printer, and robotic surgeon. Due to the delay in surgery, some patients do not have a successful outcome after treatment, especially when located at the scene of traffic accidents and on the battlefield. Imagine bringing the printer to the accident scene to perform nerve repair, which would be very timely. This technology offers a promising development for PNR. Therefore, future PNR strategies will need to be individually adapted for each patient to recreate the missing nerve structures and nerve cells.

Despite the significant advances in tissue engineering for the treatment of peripheral nerve defects, there are still many difficulties that should be overcome. For nerve defects larger than 3 cm, there is no feasible tissue engineering repair method. Despite decades of extensive research on autologous nerve substitutes, nerve autografts remain the treatment of choice for peripheral nerve deficits. The results of numerous in vivo experiments have shown that although NGCs can bridge nerve defects, the repair can barely surpass those of nerve autografts.^[^ ^23e^ ^]^ In addition, the vascular network needs to be integrated into the NGC to provide perfusion of nutrients, O_2_, and other elements. So far, only a few cells have been studied for their roles in nerve tissue engineering, and the optimal combination of biomaterials and cells remains elusive. The fabrication of structurally complex neural implants remains unachievable using current processing methods.^[^ ^27^, ^129^ ^]^ Although 3D printed NGCs have shown acceptable results in studies in vivo, the structures of conduits remain relatively simple. The main reasons for this are the poor accuracy of printer and the limited performance of bioink.

Strict FDA requirements for using this autologous nerve replacement therapy and the surgical challenges of properly welding printed structures to peripheral nerve structures remain two obstacles to the use of NGCs in the surgical field. Therefore, subsequent research in this direction should also focus on simplifying the hierarchical morphological information of nerve bundles and matching them with existing biomanufacturing techniques.^[^ ^1a^ ^]^ In the future, when imaging technology can reveal nerve bundles with the increased resolution of 3D printing technology, it can be able to use microsurgical techniques to perform the further repair of peripheral nerves at the level of nerve bundle.

In summary, significant progress has been made in recent years in preparing 3D printed NGCs with the development of effective bioinks, containing polymers, cells, and factors. Advances in technology have extended our understanding of axonal regeneration to the nanoscale. In addition, the use of 3D printing technology in regenerative medicine will eventually be developed. With advances in 3D printing resolution and medical imaging technology, NGCs can be developed in finer detail. Therefore, the ideal NGCs should be prepared by combining different fabrication techniques, using natural and synthetic polymers, and mimicking the natural nerve tissue structure. However, the perfect material for nerve repair needs to be further explored, and the transformation from quantitative to qualitative change requires long‐term efforts. Breakthroughs in related technologies and methods require interdisciplinary collaboration, such as clinical medicine, materials science, engineering, and computer technology. We believe that the combination of design, fabrication, materials, and biological factors will help us to produce bionic multifunctional NGCs that can effectively repair peripheral nerve defects clinically in the near future.

## Conflict of Interest

The authors declare no conflict of interest.
